# Supplementary material for: Netcombin: An algorithm for constructing optimal phylogenetic network from rooted triplets
Source: PLoS One. 2020 Sep 18;15(9):e0227842. doi: 10.1371/journal.pone.0227842 (PMC7500971; doi:10.1371/journal.pone.0227842)
Supplement: S4 File — (ZIP) [file pone.0227842.s004.zip › SequenceData-Triplet/sequenceData-10-20-30-0-Triplet/length 500, numseq 40, rate 10^-7/3 seq.pdf]

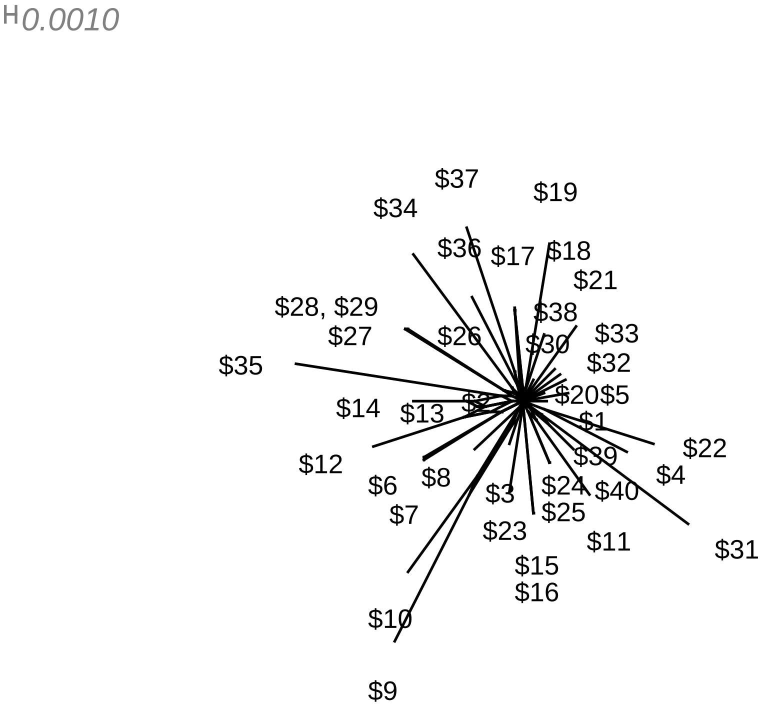
0.0010
$37
$19
$34
$36
$17
$18
$21
$28, $29
$38
$27
$26
$33
$30
$35
$32
$20
$5
$14
$2
$13
$1
$22
$12
$39
$4
$6
$8
$24
$40
$3
$7
$25
$23
$11
$31
$15
$16
$10
$9
